# Parasite Fauna and Coinfections in Urban Rats Naturally Infected by the Zoonotic Parasite *Angiostrongylus cantonensis*

**DOI:** 10.3390/pathogens13010028

**Published:** 2023-12-27

**Authors:** María Teresa Galán-Puchades, Carla Gosálvez, María Trelis, Mercedes Gómez-Samblás, Jennifer Solano-Parada, Antonio Osuna, Sandra Sáez-Durán, Rubén Bueno-Marí, Marius V. Fuentes

**Affiliations:** 1 Parasites & Health Research Group, Department of Pharmacy, Pharmaceutical Technology and Parasitology, Faculty of Pharmacy, University of Valencia, Burjassot, 46100 Valencia, Spain; carla.go.can@gmail.com (C.G.); maria.trelis@uv.es (M.T.); sandra.saez@uv.es (S.S.-D.); ruben.bueno@uv.es (R.B.-M.); mario.v.fuentes@uv.es (M.V.F.); 2 Laboratory of Biochemistry and Molecular Parasitology, Institute of Biotechnology, University of Granada, 18071 Granada, Spain; msambla@gmail.com (M.G.-S.); jesolpa@gmail.com (J.S.-P.); aosuna@ugr.es (A.O.); 3 Laboratorios Lokímica, Departamento de Investigación y Desarrollo (I+D), Ronda Auguste y Louis Lumière 23, Nave 10, Parque Tecnológico, Paterna, 46980 Valencia, Spain

**Keywords:** *Angiostrongylus cantonensis*, *Rattus norvegicus*, *Rattus rattus*, parasite fauna, coinfections

## Abstract

When the zoonotic parasite of rodents that can cause human neuroangiostrongyliasis, i.e., *Angiostrongylus cantonensis,* is found in its natural definitive hosts, it is usually reported in isolation, as if the rat lungworm were the only component of its parasite community. In this study, we report the coinfections found in rats naturally infected by *A. cantonensis* in urban populations of *Rattus norvegicus* and *Rattus rattus* in Valencia, Spain. In addition to the rat lungworms, which were found in 14 of the 125 rats studied (a prevalence of 11.20%), 18 other parasite species (intestinal and tissular protists, microsporidia and helminths) were found, some of them with high burdens. Fourteen of these nineteen species found are potential zoonotic parasites, namely *Blastocystis*, *Giardia duodenalis*, *Cryptosporidium* spp., *Enterocytozoon bieneusi*, *Encephalitozoon hellem*, *Toxoplasma gondii*, *Brachylaima* spp., *Hydatigera taeniaeformis s.l.* larvae, *Hymenolepis nana*, *Hymenolepis diminuta*, *Angiostrongylus cantonensis*, *Calodium hepaticum*, *Gongylonema neoplasticum* and *Moniliformis moniliformis*. The total predominance of coinfected rats as well as their high parasite loads seem to indicate a trend towards parasite tolerance.

## 1. Introduction

*Angiostrongylus cantonensis* (Chen, 1935) (Rhabditida: Angiostrongylidae) is a zoonotic parasite of rodents, including mainly the Norway rat, *Rattus norvegicus*, and the black rat, *Rattus rattus*, as their natural definitive hosts, which become infected through the ingestion of its intermediate hosts, snails and slugs, or its paratenic hosts, such as freshwater prawns, frogs and land crabs, among others [1]. The parasite is known as the rat lungworm, as the adults live in the pulmonary arteries of rats. When humans accidentally become infected—via the same route of transmission as rats—the parasite can cause neuroangiostrongyliasis due to the presence of the worms in the central nervous system [1]. Therefore, its control must be established under the One Health concept, as the control of zoonoses is an integral part of this approach.

*Angiostrongylus cantonensis* has been reported mainly in tropical and subtropical areas, limited by low temperatures. Until recently, the parasite seemed to be far away from Europe. However, it was found in rats and snails in Tenerife (Canary Islands, Spain) in 2010 [2]. Yet, although Tenerife is Europe in a political sense, it is Africa, geographically speaking. Several years later, in 2019, the parasite was found in hedgehogs in Mallorca (Balearic Islands, Spain) which, geographically, is Europe [3]. Therefore, it was only a matter of time before it was found in continental Europe, which our research group did in 2022 in urban/peri-urban populations of *R. norvegicus* and *R. rattus* in the city of Valencia (Spain) [4,5].

When *A. cantonensis* is found in its natural definitive hosts, to our knowledge, it is systematically reported in isolation, as if the rat lungworm were the only component of the within-rat parasite community, thus ignoring possible relationships with other parasite populations (infrapopulations) present in the infected rats. In this context, we report the first data on the concomitant parasite populations, i.e., protists, microsporidia and helminth coinfections, found in rats naturally infected by *A. cantonensis* in an urban rat population in the city of Valencia (Spain).

## 2. Materials and Methods

### 2.1. Study Area and Animals

Our research group signed an agreement with the Valencia City Council allowing us to investigate the presence of zoonotic parasites in rats trapped by the pest control company Laboratorios Lokímica, as part of the municipal pest control campaign in the city.

The presence of *A. cantonensis* was investigated in 125 rats, 97 *R. norvegicus* (43 males, 49 females and 5 indeterminate; 63 adults, 32 juveniles and 2 indeterminate) and 28 *R. rattus* (9 males and 19 females; 20 adults and 8 juveniles) trapped between April 2021 and March 2023. We studied rats captured at three trapping sites located in 17 of the 19 districts into which Valencia is divided, namely, in the sewer system (55 individuals), in city parks and gardens (43) and in orchards located in a peri-urban area of Valencia (27). Most of the rats, 74, were trapped in spring, 20 in autumn and 31 in winter. The trapped rats were kept at −20 °C until their parasitological examination.

Rat species were identified based on the external morphometry according to J. Gosàlbez [6]. Likewise, rats were considered juveniles or adults according to their body weight and external morphometry [6].

### 2.2. Parasitological and Molecular Techniques

Once thawed, the rats were dissected to extract the adult helminths from the different organs. The helminths found were studied by conventional helminthological techniques based on morphology. Cestodes and acantocephalans were stained with alcoholic hydrochloric carmine and trematodes with Grenacher’s boracic carmine, for 24 h. Subsequently, these helminths were partially destained with acidified alcohol, dehydrated in an alcohol series, cleared with xylene and mounted in Canada balsam between slide and coverslip. Nematodes were, in turn, studied by direct examination between slide and coverslip with lactophenol as clearing fluid.

The nematodes found in the pulmonary arteries were also identified by molecular techniques [4,5]. The study of protozoans in the large intestine content was made by means of the Midi Parasep^®^ SF (Apacor Ltd., Wokingham, UK) concentration technique followed by a multiplex PCR (AllplexTM Gastrointestinal Panel–Parasite Assay) for the detection of protist parasites such as *Giardia duodenalis, Entamoeba histolytica*, *Cryptosporidium* spp., *Blastocystis*, *Dientamoeba fragilis* and *Cyclospora cayetanensis* [7]. After DNA extraction, the microsporidia *Enterocytozoon bieneusi*, *Encephalitozoon intestinalis*, *E. cuniculi* and *E. hellem* were molecularly investigated by direct PCR [8]. *Toxoplasma gondii* was searched for in the rat brains by quantitative PCR (qPCR) [9] and the presence of *Leishmania infantum* in the spleens, ears and skin of the rats was also explored by qPCR [10].

### 2.3. Statistical Analysis

The comparison of the prevalences between intrinsic (age and sex) and extrinsic (site and season of capture) factors was made through the χ^2^ test. Statistical significance was established at *p* < 0.05. Results obtained in both rat species, *R. norvegicus* and *R. rattus,* were analyzed and compared.

Statistical analysis was carried out using the IBM SPSS 26.0 for Windows (International Business Machines Corporation (IBM), Armonk, NY, USA) and StatView 5.0 (Statistical Analysis System (SAS) Institute Inc., Cary, NC, USA) software packages.

## 3. Results

### 3.1. Angiostrongylus cantonensis Infected Rats

The rat lungworm was identified in 14 of the 125 captured rats (11.20%), namely in 10 *R. norvegicus* (10.31%) and in 4 *R. rattus* (14.29%). It was found in the pulmonary arteries of 13 of the studied rats (10 *R. norvegicus* and 3 *R. rattus*). One black rat also harbored juvenile parasites in the brain and another one harbored the parasites exclusively in the brain. A total of 192 individuals of *A. cantonensis* were collected in the rats, with a mean intensity of 13.71 in the infected rats.

As for the sex of the rats, all *R. norvegicus* infected by *A. cantonensis* in the present study and two *R. rattus* (12/14 (85.71%)) were male. Only the two juvenile black rats were female (2/14, 14.29%)). The presence of *A. cantonensis* in *R. norvegicus* is sex-biased, with a higher prevalence of infection in males (10/43 (23.26%)) than in females (0/49); this difference is statistically significant (χ^2^ = 12.785, *p* = 0.003). This finding is supported by the fact that in *R. rattus*, in spite of the low number of black rats parasitized (4/28), and although males (2/9 (22.22%)) are more highly parasitized than females (2/19 (10.53%)), the difference between females of both rat species is also statistically significant (χ^2^ = 5.314, *p* = 0.0212).

Concerning the age of the rats, 11 rats infected by the nematode were adults (11/83 (13.25%)) and only 3 were juveniles (3/40 (7.5%)). No statistically significant differences were found concerning the age of both rat species together. However, considering each rat species separately, juvenile black rats have a higher prevalence (2/8 (25.00%)) than juveniles of the Norwegian rats (1/32 (3.13%)); this difference is statistically significant (χ^2^ = 4.414, *p* = 0.0356).

**Figure 1:**
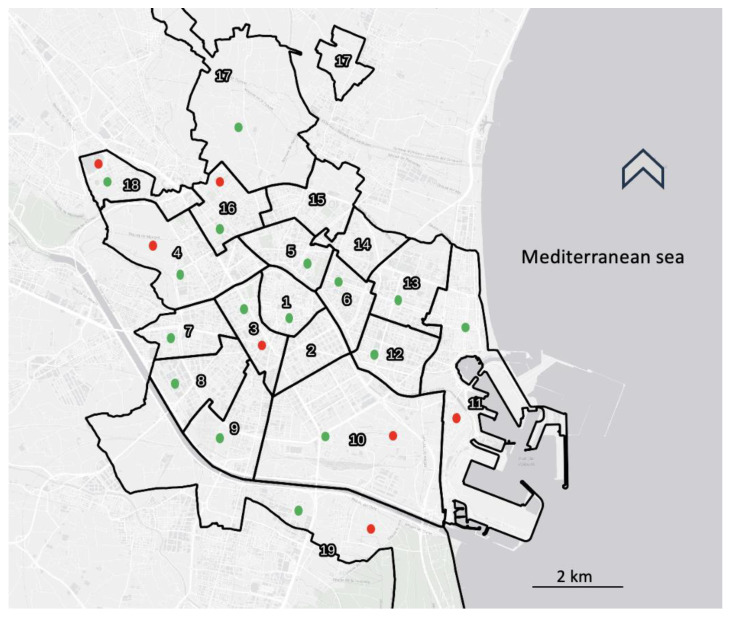
Map of the districts of Valencia; green dots showing the studied ones. *Angiostrongylus cantonensis*-infected rats were found in districts with red dots.

Angiostrongylus cantonensis was found in rats trapped in 7 of the 17 surveyed districts of Valencia (41.18%), as well as in the three trapping sites, i.e., in 6 rats of the 55 trapped in sewers (10.91%), in 2 of the 43 caught in parks (4.65%) and in 6 of the 27 captured in the orchards (22.22%) (Figure 1). Although the prevalences found are different according to the trapping sites, the results are not statistically significant when analyzed together due to the small sample sizes. However, there is a statistically significant difference between the prevalence found in parks compared to that found in orchards (χ^2^ = 5.509, *p* = 0.0245).

Of the 74 rats, 10 trapped in spring were infected by *A. cantonensis* (13.51%), while 2 were found parasitized in autumn (10%) and 2 in winter (6.45%).

### 3.2. Parasite Fauna/Coinfections

The parasite community in the studied organs of the 14 rats consisted of 19 different parasite species. Table 1 shows, in addition to *A. cantonensis*, the 18 other parasite species found according to rat species. Specifically, the parasite community of the rats studied consisted of six protists, one trematode, three cestodes, eight nematodes and one acanthocephalan. Fourteen of these nineteen species found are potentially zoonotic parasites, namely *Blastocystis*, *Giardia duodenalis*, *Cryptosporidium* spp., *Enterocytozoon bieneusi*, *Encephalitozoon hellem*, *Toxoplasma gondii*, *Brachylaima* spp., *Hydatigera taeniaeformis s.l.* larvae, *Hymenolepis nana*, *Hymenolepis diminuta*, *A. cantonensis*, *Calodium hepaticum*, *Gongylonema neoplasticum* and *Moniliformis moniliformis*. No rats were found infected by *Leishmania infantum*.

Table 2 summarizes the parasitic coinfections, i.e., the concomitant species found in the *A. cantonensis*-infected rats and their respective loads in the ten Norway rats as well as in the four black rats.

Considering the coinfections in at least more than half of the 10 *A. cantonensis*-infected *R. norvegicus*, coinfection with *C. hepaticum* was the most frequent one, as all 10 rats infected by the rat lungworm were also infected by the hepatic nematode. Coinfection with *N. brasiliensis*—both species share the lungs as a microhabitat in their life cycles—was also frequent (in 8 out of 10 rats). No other helminth coinfection occurred in more than five rats, only concomitance with the protists, *Giardia* (in seven rats) and *Blastocystis* (in six rats).

More *R. rattus* infected with *A. cantonensis* would be needed to analyze coinfections in the case of the black rat.

## 4. Discussion

### 4.1. Angiostrongylus cantonensis Infected Rats

Published prevalences of *A. cantonensis* in rats can vary from 3 to 100% depending on the endemic area [11]. In our study, we found an overall prevalence of *A. cantonenesis* of 11.20% in Valencia, which—although not very high—was obtained in highly populated zones of the city, implying a potential (probably minimal) risk of acquiring the infection in those areas, mainly parks and gardens, in which infected snails could coexist with humans and, in particular, with children in playgrounds.

Regarding the sex of the rats, *A. cantonensis* was more prevalent in males of *R. norvegicus* than in females in the studied rat population. However, depending on the study, there was no difference found in the prevalence of the rat lungworm between males and females of the Norway rat, or *A. cantonensis* was found to be even more prevalent in females than in males [12,13]. Therefore, sex-biased parasitism seems to be a complex phenomenon influenced not only by hormones but also by other additional variables [12].

Concerning the influence of the trapping season, due to the small sample sizes, the results obtained are not statistically significant.

The fact that the highest *A. cantonenesis* prevalence was found in orchards is remarkable considering the high rate of consumption of raw vegetables in the Mediterranean diet, which poses a risk of acquiring the parasite larva through the ingestion of not-sufficiently washed salads [14].

Previously, we obtained a prevalence of the rat lungworm of 8.51% when 94 rats were studied [5]. The only data on the prevalence of the nematode near Spain was obtained in Tenerife, where a prevalence of 19.19% was obtained after studying 297 rats, most of them from rural areas [15]. Therefore, although the greater the number of rats studied, the higher the prevalence, there are no statistically significant differences between these figures.

### 4.2. Parasite Fauna/Coinfections

The studied rats presented a rich and varied within-host parasite community, the most remarkable finding being that 19 infrapopulations were found in only 14 rats, 14 of these parasite species being potentially zoonotic parasites posing a possible risk of transmission to the human population with which the rats coexist.

The *A. cantonensis*-infected rats captured in the sewers presented the greatest parasite species richness, as only the stomach nematode *Mastophorus muris* and the microsporidian *E. bieneusi* were not found (Table 2).

All components of the parasite community were found in rats trapped in spring (10/14). Only two rats captured in autumn and two in winter were found to be infected by *A. cantonensis*, so the absence of certain parasites in these individuals cannot be discussed.

Except for the microsporidian *Encephalitozoon hellem,* all other parasite species, 18, were found in the 10 *R. norvegicus* infected by *A. cantonensis,* and 12 different species were found in only 4 individuals of *R. rattus* (Table 1).

Only the 10 *A.* *cantonenesis*-infected *R. norvegicus* analyzed in this study presented a richer parasite community than the 100 Norway rat individuals we previously studied in Barcelona [7,10,16], without even considering the microsporidians and *T. gondii*, which were not investigated in the Barcelona rats.

Concerning coinfections, there was no case of monoparasitism among the studied rats. Adult *A. cantonensis*-infected *R. norvegicus* harbored from 6 to 12 different species in the same individual. The case of one adult *R. norvegicus* that harbored representatives of protists, trematodes, cestodes, nematodes as well as acanthocephalans in the intestine, with most of the helminths having high parasite loads (Table 2, *Rn* IX), is remarkable. Also noteworthy is the case of one juvenile Norway rat harboring 57 individuals of *A. cantonensis* (26 males and 31 females) in the pulmonary arteries as well as bearing a high burden of *C. hepaticum* infecting the liver (Table 2, *Rn* X). Both rats were trapped in the sewer system.

When analyzing the parasite community/coinfections found and the transmission routes, in the case of the monoxenous protists and microsporidia, rats became infected by the fecal/oral transmission route directly throughout the ingestion of cysts/oocysts/spores contaminating the environment, in particular the sewer system, and orchards, which are not normally irrigated with safe or potable water.

The presence of *T. gondii* in the rats may also be related to contamination by oocysts from cat feces or by cannibalism, a common occurrence in cases of limited food supply. The absence of the usual amount of food on the streets, due to the lockdown and the closure of restaurants during the pandemic, could have led to an increase in cannibalism that favored the *T. gondii* life cycle.

Considering the helminth parasites, 7 worms presented an indirect or heteroxenous life cycle and 5 had a monoxenous or direct cycle (Table 1). In the case of *H. nana,* the parasite is able to complete its life cycle either with the intervention of an arthropod intermediate host harboring the larval stage (cysticercoid) (heteroxenous life cycle) or without the intervention of any intermediate host but directly inside the intestine of the definitive host (monoxenous life cycle). Only one *R. norvegicus* presented a high *H. nana* load (*R.n.* IX in Table 2), which suggests the monoxenous-type cycle.

Among the monoxenous helminths, eggs shed in feces (or urine in the case of *Trichosomoides crassicauda*) are infective for the rats once the eggs embryonate in the soil. To become infected by *C. hepaticum*, a nematode that lives in the liver parenchyma, rats must also ingest the eggs that contaminate the environment. However, in this case, as the eggs are trapped in the liver, the rat must die in order to release the eggs, which mature in the soil. It is noteworthy that all the 10 *R. norvegicus* were infected by *C. hepaticum* (Table 2). This could indicate an increase in rat mortality during the pandemic period that ultimately favored cannibalism, leading to the release of eggs into the environment, enhancing the life cycle of *C. hepaticum*, as in the case of *T. gondii.*

In the case of *Nippostrongylus brasiliensis* (a murine model of *Necator americanus*), the larvae penetrate the skin of rats, or may also be ingested from the soil, and after molting and maturing in the lungs, reach the small intestine. The eggs are released in feces and hatch in the soil, releasing the L1 larvae, which become infective after molting. The nematode was found in almost all Norway rats at high burden levels (Table 2). Exceptionally, two of them (VIII and IX) harbored hundreds and hundreds of *N. brasiliensis*. It is hard to believe that this life cycle does not include processes of autoinfection and that the extraordinary number of adults in the intestine is due to repeated infections.

For heteroxenous life cycles, rats must ingest the eggs of *Hydatigera taeniaeformis* shed in cat (definitive host) feces. Rats act as intermediate hosts harboring the metacestode (strobylocercus) in the liver parenchyma. Cats are the only predator that rats have in cities, completing the biological cycle. Several rats harbored both *H. taeniaeformis* and *T. gondii*, parasites that share a common infection route, i.e., cat feces.

To become infected by *Brachylaima* spp. and *A. cantonensis,* rats must ingest infected snails (also slugs or paratenic hosts in the case of the rat lungworms). Two Norway rats were coinfected by both helminths, leading to the hypothesis that these two parasites could have shared a snail as an intermediate host.

For the rest of the heteroxenous helminths, intermediate hosts involve arthropods, mainly beetles for *H. diminuta* and cockroaches for *M. muris, G. neoplasticum* and *M. moniliformis*, with arthropods being an important element of the rat diet.

In terms of the host microhabitats for which the worms might compete, *A. cantonenesis* (adults, eggs and L1 larvae) and *N. brasiliensis* larvae (L3 and L4) share the same microhabitat, i.e., the lungs. In this regard, 8 of the 10 *R. norvegicus* and 1 *R. rattus* harbored both species (Table 2), so they do not appear to be competitors, at least in the studied rats. Likewise, six rats harbored *A. cantonensis* and *T. gondii*, parasites that share the brain as a microhabitat at a particular time of their life cycles.

The liver was also coinfected by the tapeworm larvae of *H. taeniaeformis* and *C. hepaticum* in three *R. norvegicus*, while the small intestine presented the greatest species richness, namely up to five different ones (Table 1). *Nippostrongylus brasiliensis* always occupies the first part of the small intestine, the duodenum, while the remaining helminths (*Hymenolepis* spp., *Brachylaima* spp. and *M. moniliformis*) are usually located in the jejunum and ileum.

Hosts which are ubiquitous, like rats, are more likely to become coinfected, as are hosts that occupy different ecological niches in which several parasites are present [17]. Consequently, rats that flourish in a wide range of environmental conditions, like sewers, parks, gardens and orchards in this particular case, are exposed to a greater diversity of parasites. Once infected, hosts use two strategies to cope with their parasites: resistance or tolerance [18]. Hosts can, by different mechanisms, reduce parasite burdens (resistance) or they can minimize the damage caused by the parasite load (tolerance). *Angiostrongylus cantonensis* was experimentally shown to cause a 10–20% mortality in *R. norvegicus* [19]. Also, an experimental study on parasite tolerance showed that mortality is related to the number of larvae of the rat lungworm used to infect rats [20]. However, it is difficult to know how to extrapolate these findings based on laboratory rats—not infected by any other parasite—to understand the consequences of coinfection in nature. In addition, coinfections can have negative effects on the host, accelerating its mortality or, otherwise, coinfections can have positive effects on the host, reducing its mortality [21].

## 5. Conclusions

Although we are aware of the limited number of rats studied, and considering that no histopathological studies were carried out to assess possible tissue damage, the total predominance of coinfected rats as well as their high parasite loads seem to indicate a trend towards parasite tolerance, at least in the studied rats infected by *A. cantonensis*. In this context, it seems clear that if at some point coinfections led to an increase in the mortality rate of the urban rat populations in Valencia, those populations that survived, considering their high reproductive capacity, may have given rise to tolerant populations that justify these high prevalences, parasite loads, and coinfections found in this study. In any case, further studies covering larger rat populations over the years as well as histopathological studies will help to determine whether tolerance is, in fact, the strategy that rat populations have developed against their parasites.

## Figures and Tables

**Table 1:** Parasite species found in 10 *Rattus norvegicus* and 4 *Rattus rattus* in urban and peri-urban areas of Valencia, Spain, captured between 2021 and 2023.

Protists/Microsporidia Species	Microhabitat	Cycle	n (Host)	P (%)
*Blastocystis*	small intestine	M	8 (6 *Rn*, 2 *Rr*)	60 *Rn* 50 *Rr*
*Giardia duodenalis*	small intestine	M	9 (7 *Rn*, 2 *Rr*)	70 *Rn* 50 *Rr*
*Cryptosporidium* spp.	small intestine	M	1 (1 *Rn*)	10 *Rn*
*Enterocytozoon bieneusi*	small intestine	M	2 (1 *Rn*, 1 *Rr*)	10 *Rn* 25 *Rr*
*Encephalitozoon hellem*	small intestine	M	1 (1 *Rr*)	25 *Rr*
*Toxoplasma gondii*	brain	D	6 (5 *Rn*, 1 *Rr*)	50 *Rn* 25 *Rr*
**Helminth species**				
*Brachylaima* spp.	small intestine	H	1 (1 *Rn*)	10 *Rn*
*Hydatigera taeniaeformis s.l.* larvae	liver	H	5 (4 *Rn*, 1 *Rr*)	40 *Rn* 25 *Rr*
*Hymenolepis nana*	small intestine	H/M	3 (3 *Rn*)	30 *Rn*
*Hymenolepis diminuta*	small intestine	H	4 (3 *Rn*, 1 *Rr*)	30 *Rn* 25 *Rr*
*Angiostrongylus cantonensis*	pulmonary arteries/brain	H	14 ^a^ (10 *Rn*, 4 *Rr*)	100 *Rn* 100 *Rr*
*Calodium hepaticum* ^b^	liver	M	11 (10 *Rn*, 1 *Rr*)	100 *Rn* 25 *Rr*
*Mastophorus muris*	stomach	H	2 (1 *Rn*, 1 *Rr*)	10 *Rn* 25 *Rr*
*Eucoleus gastricus*	stomach	M	3 (3 *Rn*)	30 *Rn*
*Trichosomoides crassicauda*	urinary bladder	M	3 (3 *Rn*)	30 *Rn*
*Nippostrongylus brasiliensis*	small intestine/ lungs (larvae)	M	9 (8 *Rn*, 1 *Rr*)	80 *Rn* 25 *Rr*
*Heterakis spumosa*	large intestine	M	4 (3 *Rn*, 1 *Rr*)	30 *Rn* 25 *Rr*
*Gongylonema neoplasticum*	esophagus/ stomach	H	2 (2 *Rn*)	20 *Rn*
*Moniliformis moniliformis*	small intestine	H	2 (2 *Rn*)	20 *Rn*

^a^ In pulmonary arteries in 10 *Rn* and 3 *Rr*; in pulmonary arteries and brain in 1 *Rn* and only in brain in 1 *Rr.* ^b^ Range for *C. hepaticum* is not reported due to the difficulty in the reconstruction of dead parasites. Abbreviations: *Rn*, *Rattus norvegicus*; *Rr*, *Rattus rattus*; H, heteroxenous; M, monoxenous; n, number of parasitized hosts; P, prevalence. Potentially zoonotic species shaded in grey.

**Table 2:** Concomitant parasite species in naturally infected *Rattus norvegicus* (*Rr*) and *R. rattus* (*Rr*) by *Angiostrongylus cantonensis*: numbers represent helminth loads, and the total number of parasite species in individual rats is written in bold.

Protists/ Microsporidia Species	*Rn* I ^s^ */Rr* * I ^s^	*Rn* II ^s^ */Rr* II ^p^	*Rn* III ^o^ /*Rr* III ^o^	*Rn* IV ^o^ /*Rr* * IV ^o^	*Rn* V ^o^	*Rn* VI ^o^	*Rn* VII ^p^	*Rn* VIII ^s^	*Rn* IX ^s^	*Rn* * X ^s^
*Blastocystis*	+/+	+/−		−/+		+		+	+	+
*G. duodenalis*	+/+	+/+		+/−	+		+	+	+	
*Cryptosporidium* spp.									+	
*T. gondii*	+/−		−/+		+	+	+	+		
*E. hellem*	−/+									
*E. bieneusi*				−/+		+				
**Helminth species**										
*Brachylaima* spp.				4/−					4	
*H. t. s.l.* larvae	1/−	−/1	1/−					1		
*H. nana*								1	65	
*H. diminuta*		2/−	6/2	2/−		2	43			
*A. cantonensis*	2/30	4/9	7/3	13/2	7	3	33	4	19	57
*C. hepaticum*	+/−	+/−	+/+	+/−	+	+	+	+	+	+
*M. muris*			10/2			2				
*E. gastricus*				5/−			9		21	
*T. crassicauda*				4/−	4				14	
*N. brasiliensis*	80/−		65/2	31/−	6	2	25	HH	HH	
*H. spumosa*	9/−			2/7	1					
*G. neoplasticum*				1/−					3	
*M. moniliformis*		2/−							7	
**Total n° species**	**8**	**6**	**6**	**10**	**7**	**8**	**7**	**8**	**12**	**3**

s, Sewers; o, orchards; p, parks. *—juvenile. Abbreviations: *G. duodenalis*, Giardia duodenalis; *T. gondii*, Toxoplasma gondii; *E. hellem*, Encephalitozoon hellem; *E. bieneusi*, Enterocytozoon bieneusi; H. t. s.l. larvae, Hydatigera taeniaeformis s.l. larvae; *H. nana*, Hymenolepis nana; *H. diminuta*, Hymenolepis diminuta; *A. cantonensis*, Angiostrongylus cantonensis; *C. hepaticum*, Calodium hepaticum; *M. muris*, Mastophorus muris; *E. gastricus*, Eucoleus gastricus; *T. crassicauda*, Trichosomoides crassicauda; *N. brasiliensis*, Nippostrongylus brasiliensis; *H. spumosa*, Heterakis spumosa; *G. neoplasticum*, Gongylonema neoplasticum; *M. moniliformis*, Moniliformis moniliformis; HH, hundreds. + Numbers are not reported in *C. hepaticum* due to the difficulty in the reconstruction of dead parasites. Potentially zoonotic species shaded in grey.

## Data Availability

No new data were created or analyzed in this study. Data sharing is not applicable to this article.
